# Supplementary material for: Predation and fragmentation portrayed in the statistical structure of prey time series
Source: BMC Ecol. 2009 May 6;9:10. doi: 10.1186/1472-6785-9-10 (PMC2689204; doi:10.1186/1472-6785-9-10)
Supplement: Additional file 2 — Voles and related classes ODDox Documentation. ODDox documentation of the agent-based model (ALMaSS) applied by Hendrichsen et al. The documentation is started by activating main.html. [file 1472-6785-9-10-S2.zip › Vole_ODDox/_winter_wheat_8_h.html]

ALMaSS ODDox: WinterWheat.H File Reference

- Main Page
- Related Pages
- Classes
- Files

# WinterWheat.H File Reference

---

## Detailed Description

**WinterWheat.h This file contains the headers for the WinterWheat class**   

by Chris J. Topping   
Version of June 2003   
All rights reserved.   
  
With additions as noted in:   
Doxygen formatted comments in July 2008   

|  |  |
| --- | --- |
|  |  |
| Classes | |
| class | WinterWheat |
|  | WinterWheat class   . More... |
| Defines | |
| #define | WINTERWHEAT\_BASE   5100 |
| #define | WW\_AUTUMN\_PLOUGH   m\_field->m\_user[1] |
|  | A flag used to indicate autumn ploughing status. |
| Enumerations | |
| enum | WinterWheatToDo {     ww\_start = 1, ww\_sleep\_all\_day = WINTERWHEAT\_BASE, ww\_ferti\_p1, ww\_ferti\_s1,     ww\_ferti\_s2, ww\_autumn\_plough, ww\_autumn\_harrow, ww\_stubble\_harrow1,     ww\_autumn\_sow, ww\_autumn\_roll, ww\_ferti\_p2, ww\_herbicide1,     ww\_spring\_roll, ww\_herbicide2, ww\_GR, ww\_fungicide,     ww\_fungicide2, ww\_insecticide1, ww\_insecticide2, ww\_insecticide3,     ww\_strigling1, ww\_strigling2, ww\_water1, ww\_water2,     ww\_ferti\_p3, ww\_ferti\_p4, ww\_ferti\_p5, ww\_ferti\_s3,     ww\_ferti\_s4, ww\_ferti\_s5, ww\_harvest, ww\_straw\_chopping,     ww\_hay\_turning, ww\_hay\_baling, ww\_stubble\_harrow2, ww\_grubning   } |

---

## Define Documentation

#define WINTERWHEAT\_BASE   5100

#define WW\_AUTUMN\_PLOUGH   m\_field->m\_user[1]

A flag used to indicate autumn ploughing status.

Referenced by WinterWheat::Do().

---

## Enumeration Type Documentation

enum WinterWheatToDo

Below is the list of things that a farmer can do if he is growing winter wheat, at least following this basic plan. So all we have to do is figure out when to do the different things. Once we have done some kind of management, then an event is triggered and the fact that this particular management is done is registered with the particular polygon. This information is available for any ALMaSS components to inspect - e.g. animals & birds

**Enumerator:**
:   |  |  |
    | --- | --- |
    | *ww\_start* |  |
    | *ww\_sleep\_all\_day* |  |
    | *ww\_ferti\_p1* |  |
    | *ww\_ferti\_s1* |  |
    | *ww\_ferti\_s2* |  |
    | *ww\_autumn\_plough* |  |
    | *ww\_autumn\_harrow* |  |
    | *ww\_stubble\_harrow1* |  |
    | *ww\_autumn\_sow* |  |
    | *ww\_autumn\_roll* |  |
    | *ww\_ferti\_p2* |  |
    | *ww\_herbicide1* |  |
    | *ww\_spring\_roll* |  |
    | *ww\_herbicide2* |  |
    | *ww\_GR* |  |
    | *ww\_fungicide* |  |
    | *ww\_fungicide2* |  |
    | *ww\_insecticide1* |  |
    | *ww\_insecticide2* |  |
    | *ww\_insecticide3* |  |
    | *ww\_strigling1* |  |
    | *ww\_strigling2* |  |
    | *ww\_water1* |  |
    | *ww\_water2* |  |
    | *ww\_ferti\_p3* |  |
    | *ww\_ferti\_p4* |  |
    | *ww\_ferti\_p5* |  |
    | *ww\_ferti\_s3* |  |
    | *ww\_ferti\_s4* |  |
    | *ww\_ferti\_s5* |  |
    | *ww\_harvest* |  |
    | *ww\_straw\_chopping* |  |
    | *ww\_hay\_turning* |  |
    | *ww\_hay\_baling* |  |
    | *ww\_stubble\_harrow2* |  |
    | *ww\_grubning* |  |

```
00065              {
00066   ww_start = 1, // Compulsory, must always be 1 (one).
00067   ww_sleep_all_day = WINTERWHEAT_BASE,
00068   ww_ferti_p1,
00069   ww_ferti_s1,
00070   ww_ferti_s2,
00071   ww_autumn_plough,
00072   ww_autumn_harrow,
00073   ww_stubble_harrow1,
00074   ww_autumn_sow,
00075   ww_autumn_roll,
00076   ww_ferti_p2,
00077   ww_herbicide1,
00078   ww_spring_roll,
00079   ww_herbicide2,
00080   ww_GR,
00081   ww_fungicide,
00082   ww_fungicide2,
00083   ww_insecticide1,
00084   ww_insecticide2,
00085   ww_insecticide3,
00086   ww_strigling1,
00087   ww_strigling2,
00088   ww_water1,
00089   ww_water2,
00090   ww_ferti_p3,
00091   ww_ferti_p4,
00092   ww_ferti_p5,
00093   ww_ferti_s3,
00094   ww_ferti_s4,
00095   ww_ferti_s5,
00096   ww_harvest,
00097   ww_straw_chopping,
00098   ww_hay_turning,
00099   ww_hay_baling,
00100   ww_stubble_harrow2,
00101   ww_grubning,
00102 } WinterWheatToDo;
```

---

Generated on Thu Jan 22 14:13:45 2009 for ALMaSS ODDox by 
 1.5.6
